# Elucidation of Operon Structures across Closely Related Bacterial Genomes

**DOI:** 10.1371/journal.pone.0100999

**Published:** 2014-06-24

**Authors:** Chuan Zhou, Qin Ma, Guojun Li

**Affiliations:** 1 School of Mathematics, Shandong University, Jinan, China; 2 Computational Systems Biology Laboratory, Department of Biochemistry and Molecular Biology and Institute of Bioinformatics, University of Georgia, Athens, Georgia, United States of America; University of Florida, United States of America

## Abstract

About half of the protein-coding genes in prokaryotic genomes are organized into operons to facilitate co-regulation during transcription. With the evolution of genomes, operon structures are undergoing changes which could coordinate diverse gene expression patterns in response to various stimuli during the life cycle of a bacterial cell. Here we developed a graph-based model to elucidate the diversity of operon structures across a set of closely related bacterial genomes. In the constructed graph, each node represents one orthologous gene group (OGG) and a pair of nodes will be connected if any two genes, from the corresponding two OGGs respectively, are located in the same operon as immediate neighbors in any of the considered genomes. Through identifying the connected components in the above graph, we found that genes in a connected component are likely to be functionally related and these identified components tend to form treelike topology, such as paths and stars, corresponding to different biological mechanisms in transcriptional regulation as follows. Specifically, (i) a path-structure component integrates genes encoding a protein complex, such as ribosome; and (ii) a star-structure component not only groups related genes together, but also reflects the key functional roles of the central node of this component, such as the ABC transporter with a transporter permease and substrate-binding proteins surrounding it. Most interestingly, the genes from organisms with highly diverse living environments, i.e., biomass degraders and animal pathogens of clostridia in our study, can be clearly classified into different topological groups on some connected components.

## Introduction

Operons are basic transcription units in prokaryotic genomes and genes in an operon tend to be transcribed into a single mRNA and have related biological functions [1]–[3]. Operons undergo lots of changes in their content during evolution [4], [5], which results in different operon structures across multiple organisms. Only a few operons are known to be conserved across distantly related organisms [3], [6]–[8], which could be used for making functional inferences. Since more and more genomes have been completely sequenced and are accessible publicly, substantial amount of operons are predicted by high-accuracy programs [9]–[14] and are organized into well-maintained databases [15]–[19], such as DOOR2.0, which contains predicted operons for more than 2,000 prokaryotic genomes.

As proposed by Price MN [7], both operon creation and destruction could lead to large changes in gene expression patterns. Efficiently predicting conserved operons and analyzing their structures across a set of genomes can give us valuable clues to the functions and expression patterns of involved genes. Genomic co-localized gene pairs, which is a key factor in the prediction of operons [12], [13], [17], are used to analyze operon conservation across a set of organisms [7], [20]. However, the information alone could not capture the overall structural changes of a group of functionally related genes. For example, even though such a gene pair is identified in several operons from different organisms, these operons may have different structures by gaining or losing new genes due to specific requirements in transcriptional regulation [7]. Meanwhile, various similarity scores are defined between operons from different organisms [13]–[16] and could be used to identify conserved operon groups, however, they cannot decipher the complex operon topological linkages across a set of bacterial genomes.

In this paper, using identified 41,757 orthologous gene groups (OGGs) of 40 clostridial genomes [21], we integrated operon structures from 19 clostridial genomes belonging to 19 species respectively into a graph-based model, named *operon alignment graph*. Furthermore, we identified *connected operon components* (COCs) in this graph, which represent clusters of genes supported by the operon structures in at least two genomes in their pair-wise relationship. To the best of knowledge, we are the first to elucidate operon structures in this way and we have found that (i) the operon alignment graph are sparsely connected; (ii) genes in the same COC usually share similar biological functions, such as same metabolic or regulatory pathways; and (iii) different operon linkage patterns emerge in identified COCs, which corresponds to different relationships among the underlying genes.

## Materials and Methods

### Data

We downloaded 40 fully sequenced clostridial genomes from NCBI GenBank [22] as of December 2012, and their operons were retrieved from the DOOR2.0 database [15] (we only consider operons containing more than one genes). Out of these 40 organisms, 13 are biomass degraders [23]–[28], 21 are pathogens [29]–[35] and six are less characterized other kind [29], [36], [37]. Since above 40 genomes belong to 19 species, we selected one representative genome from each species (see Table 1 for details). A total of 41,738 OGGs were predicted using our in-house program GOST [38] following by the clustering tool MCL [39]. The ID for each OGG is assigned as its ranking in the output of MCL. It is worth noting that, in different OGGs, the ratio of genes between biomass degraders and pathogens varies and relative details can be found in File S1.

**Table 1:** The 19 clostridial organisms for constructing the operon alignment graph.

ID	Type	Organism	#gene	#multi-gene operon	#gene in multi-gene operon
**1**	**B**	thermocellum_ATCC_27405_uid57917	3173	596	1794
**2**	**B**	beijerinckii_NCIMB_8052_uid58137	5020	872	2378
**3**	**B**	phytofermentans_ISDg_uid58519	3902	722	1950
**4**	**B**	cellulolyticum_H10_uid58709	3390	678	2146
**5**	**B**	saccharolyticum_WM1_uid51419	4154	893	2874
**6**	**B**	cellulovorans_743B_uid51503	4254	796	2279
**7**	**B**	lentocellum_DSM_5427_uid49117	4182	842	2717
**8**	**B**	clariflavum_DSM_19732_uid82345	3892	763	2157
**9**	**B**	BNL1100_uid84307	3920	812	2610
**10**	**B**	acetobutylicum_EA_2018_uid159515	3916	757	2346
**11**	**P**	perfringens_13_uid57681	2723	490	1433
**12**	**P**	tetani_E88_uid57683	2439	512	1558
**13**	**P**	difficile_630_uid57679	3739	760	2335
**14**	**P**	botulinum_H04402_065_uid162091	3691	721	2049
**15**	**O**	novyi_NT_uid58643	2315	444	1432
**16**	**O**	kluyveri_DSM_555_uid58885	3913	766	2356
**17**	**O**	ljungdahlii_DSM_13528_uid50583	4184	848	2530
**18**	**O**	sticklandii_DSM_519_uid59585	2573	517	1908
**19**	**O**	SY8519_uid68705	2613	545	1750

**B** for biomass degrader, **P** for animal pathogen, and **O** for the others.

### Construction of operon alignment graph

Firstly, we introduce some terminologies in graph theory, which will be used in the following model. A directed graph ***D*** consists of a non-empty node set, *V*(***D***), and an edge set, *A*(***D***), connecting ordered pairs of nodes. For an edge (*u*, *v*), *u* is its tail and *v* is its head and the two nodes are called adjacent. A node is incident to an edge *e* if it is the head or tail of the edge. The degree of a node is the number of edge incident with it. Without considering the direction of edges, a connected component of ***D*** is a maximal sub-graph in which any pair of nodes is connected by at least one path and if a connected graph ***D*** doesn't contain a cycle, it's called a tree [40].

**Figure 1:**
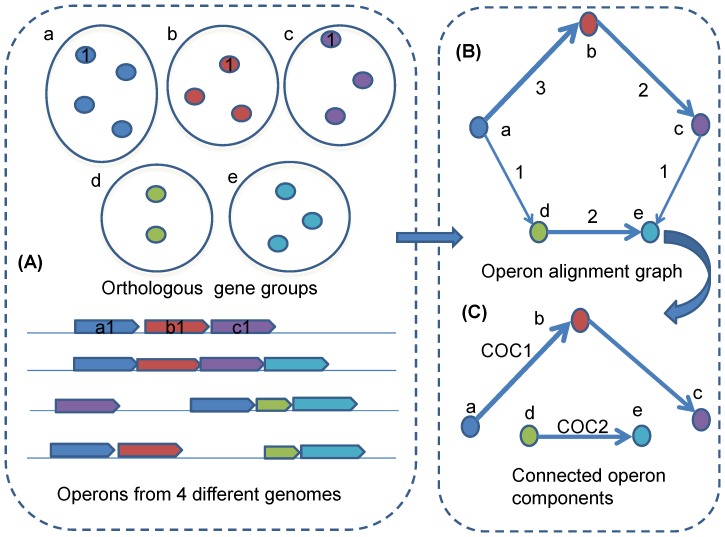
Methodology outline. (A) Five orthologous gene groups and operons from four different genomes are given as an example; (B) Operon alignment graph with edge weight indicating the number operon pairs can be aligned to the edge; (C) The connected operon components are identified by removing the edges of weight one.

We defined an operon alignment graph *G* as a directed graph based on 19 clostridial genomes, with each node representing an OGG and a pair of nodes being connected by an edge if a pair of genes, from the two corresponding OGGs respectively, was immediate neighbors in an operon in at least one genome. Intuitively, an operon should correspond to a directed path in this graph as the single-gene operons are excluded in our study (see Figure 1). Specifically, considering three OGGs *a*, *b* and *c*, where genes *a_1_*, *b_1_* and *c_1_* were from these three groups respectively, if these three genes formed an operon *A* in a specific genome following the order *a_1_*-*b_1_*-*c_1_* along the genome, we added two edges (*a*, *b*) and (*b*, *c*) in the operon alignment graph, and the gene pair (*a_1_*, *b_1_*) is called being mapped to edge (*a*, *b*) and operon *A* is called being mapped to the path (*a*, *b*, *c*). It is worth noting that the edge (*a*, *c*) will not be created as *a_1_* and *c_1_* are not consecutively located along the genome. The weight of an edge was defined as the number of gene pairs mapped to this edge, as multiple gene pairs could be mapped to the same edge when multiple genomes are considered in the model construction. After all gene pairs were added, we removed all isolated nodes (don't incident with any edge) in the current graph, which led to the final operon alignment graph *G*.

### Identification of connected operon components

In an operon alignment graph, the COCs were identified through removing the edges of weight one, which were considered to be not conserved in our model. Obviously, a COC is composed by a set of OGGs, whose genes prefer to stay in same operons across multiple related genomes. Here we only considered the COCs containing at least two OGGs. The conservation score of a COC was defined as the average weight of all its edges. Intuitively, the more conserved a COC is, the more operons were mapped to it. For example, the two COCs 1 and 2 in Figure 1 have conservation scores 2.5 and 2, respectively. We sorted all identified COCs in the decreasing order of the number of component OGGs, and used this ranking index as the ID of corresponding COC.

### Functional enrichment analysis for COCs

For the nodes of a COC, we could do functional enrichment analysis of the corresponding genes with DAVID [41]. More specifically, given a set of OGGs, we picked their genes from a certain genome as templates, such as *Clostridium thermocellum* (*C. thermocellum*), which will be submitted to DAVID as the input gene list with this genome as background genome. The *p*-values were calculated in terms of a Bonferroni-corrected modified Fisher's exact test under the null hypothesis that this set of genes was not enriched with certain biological functions.

### *Cis-regulatory* motif analysis for COCs

The *cis*-regulatory motif analyses were done with the BoBro2.0 toolkit [42], [43] and a DNA motif analysis web server DMINDA [44]. For a specific COC, we collected all the leading genes of the involved operons, then picked the upstream intergenic regions of these genes as promoter sequences, with length at most 300 bps. In this study, we were particularly interested in biomass degraders and animal pathogens. Hence, the *de-novo* motif finding and motif comparison analyses were carried out regarding these two promoter groups [42], [45].

## Results

### Construction of operon alignment graph of 19 clostridial genomes

**Figure 2:**
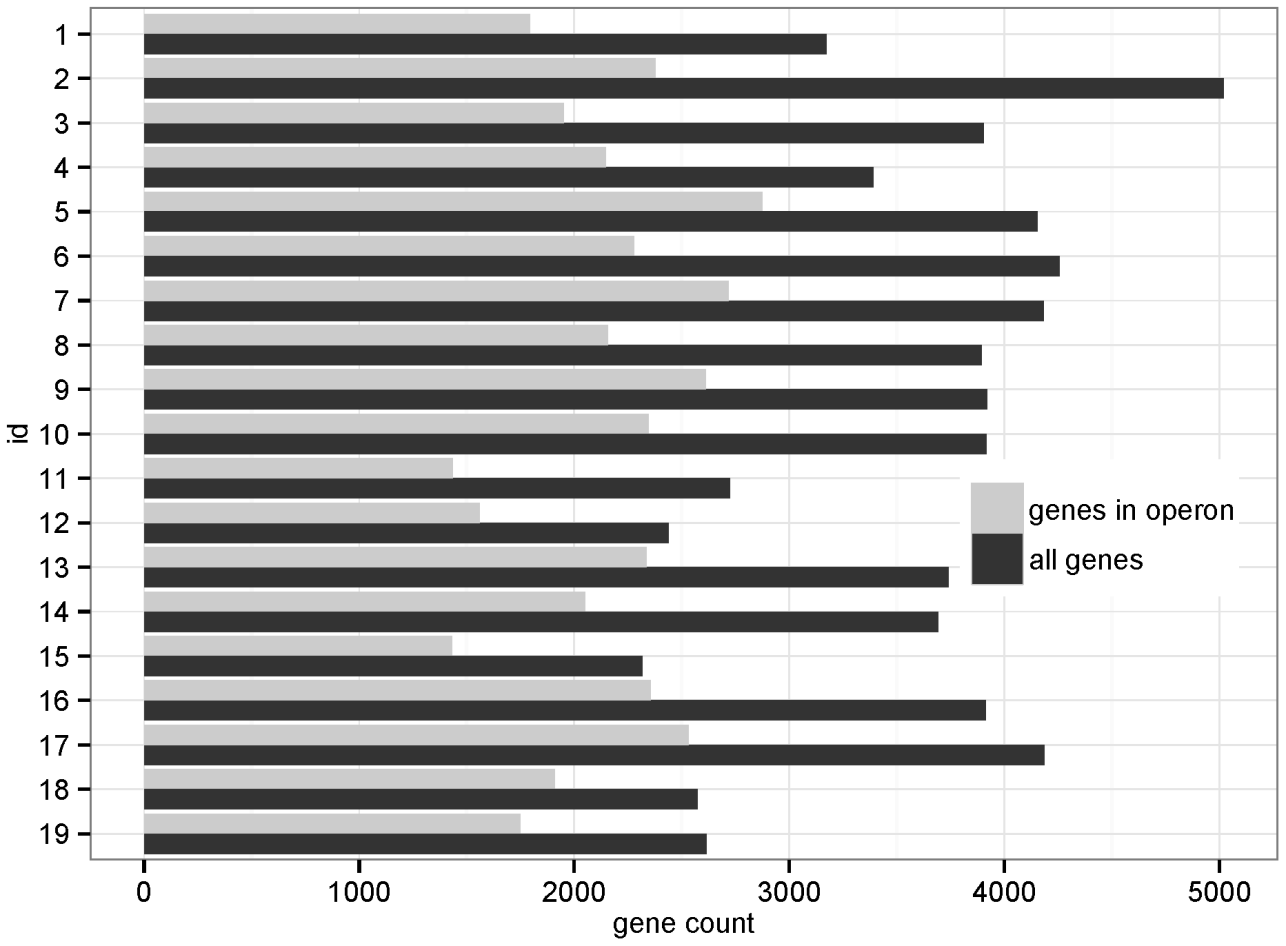
Gene count and in-operon ratio for each organism. Genome IDs are listed in Table 1 and the operons are retrieved from DOOR2.0 database.

In the 19 clostridial genomes, from 47% to 74% genes are in multi-gene operon. See Figure 2 and Table 1 for details. The operon alignment graph, constructed using these genomes, contains 22,026 nodes (about 61.7% of all OGGs), 18,924 edges and forms 4,383 connected components. The largest component contains 6,350 OGGs and 7,275 edges (see Figure S1), and each of other components contains less than 400 OGGs. About 82% of edges are of weight one in this graph (Figure S2), which means that only one operon could be mapped to that edge. We suspect that such non-conserved relationship may be newly formed according to diverse living environment of Clostridia. These results show that the operon alignment graph is sparsely connected (the number of nodes is even larger than that of edges) and genes only tend to group with specific members through the operon linkage, which is consistent with the fact that operons often encode functionally linked proteins.

While the degrees of most of the nodes (97.7%) are less than six, only 117 nodes have degrees larger than ten (Figure S3). Such large-degree property of these nodes suggests that the genes in these orthologous groups tend to form operons with various kinds of genes which are involved in diverse biological functions. Functional analysis with DAVID shows that nucleoside binding proteins are significantly enriched in the large-degree gene set (*p*-value 5e-3), indicating that they can functionally work together with different kinds of proteins (see details in file S2).

### Most of COCs adapt a tree structure and have a main functional theme

**Figure 3:**
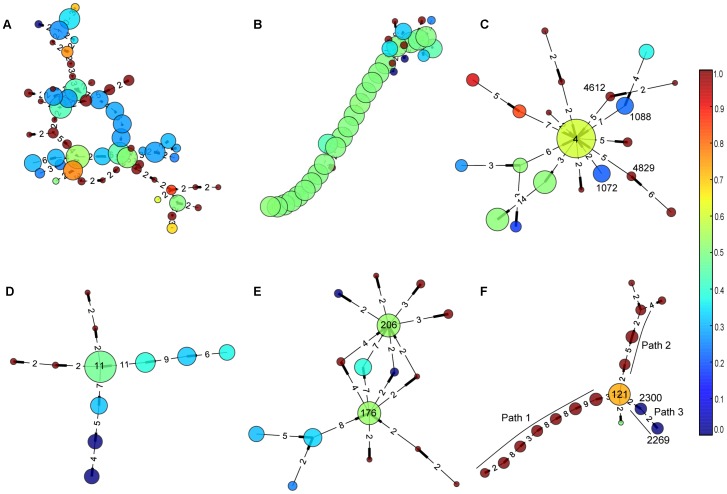
Six typical connected operon components. The size of node is proportional to the number of genes in corresponding orthologous gene group, the larger the more genes. The color indicates the proportion of genes from biomass degraders or pathogens in this group, where red color means more biomass-degrader genes while blue color represents more pathogen genes. The weights of edges are shown as numbers on the components. COC #1 in (A) is the largest COC, which contains 58 nodes, most of the genes are related to porphyrin metabolism; COC #6 in (B) contains a long path structure and mainly contains ribosomal proteins; COC #13, #54, #29 in (C), (D) and (E) respectively form the star structure; and COC #27 in (F) shows the biomass-degrader genes and pathogen genes as different topological parts.

While the operon alignment graph gives us a global view of operon linkage patterns, we use COC to describe conserved operon connectivity among genes. We identified 157 COCs containing more than five OGGs, and 63% of them were trees; while 98% of all other COCs were trees. These tree structures are consistent with the sparseness of the operon alignment graph. We observed that genes in each COC usually had a main functional theme (the top eight COCs are listed in Table 2). As we show in the following examples, COCs can efficiently group functional related genes together and be used to infer unknown gene functions. In Figure 3, we showcased some COCs, where node size is proportional to the number of genes in the OGG, the larger the more genes, color indicates the percentage of biomass degrader genes, red for more biomass degrader genes and blue for more pathogen genes, and the weights of edges are shown as numbers. More details can be found in File S3 and S4.

**Table 2:** COCs have a main functional theme through gene enrichment analysis.

COC id	#node	#edge	Edge average weight	Node maximum degree	Functional annotation from DAVID	enrichment score
**1**	58	66	3.09	7	porphyrin metabolic process	8.54
**2**	52	54	3.87	7	pyrimidine biosynthesis	4.41
**3**	51	51	4.9	5	Taxis	9.22
**4**	41	44	4.52	8	rRNA processing	1.84
**5**	40	44	3.18	8	nucleotide catabolic process	2.89
**6**	36	43	9.93	8	ribosomal protein	22.64
**7**	29	33	2.88	9	*	*
**8**	25	24	2.25	4	metal ion binding	2.67

(*) no cluster identified.

The largest COC contains 58 OGGs (Figure 3A). The DAVID analysis shows that, for the subset of genes contained in *C. thermocellum*, one functional cluster (enrichment score 8.54) contains about 73% of all genes (*p*-value 1.02e-11); and the GO TERM annotations suggest that these genes are mainly involved in porphyrin metabolic and biosynthetic process. Meanwhile for genes in Clostridium difficile 630 (C. difficile), one functional cluster (enrichment score 21.89, *p*-value 3.62e-29) contains more than 85% of all genes, which are related to porphyrim metabolic process and biosynthetic.

We have also identified 46 COCs with a simple path structure, which is an extremely simplified tree, such as COC #6 (Figure 3B) with 36 nodes and average weight as high as 9.93. DAVID analysis suggests that 81% of genes from C. thermocellum (enrichment score 22.6, *p*-value 3.00e-34) and 85% from C. difficile (enrichment score 24.3, *p*-value 1.07e-38) in COC #6 mainly correspond to ribosomal proteins. More detailed analysis with NCBI annotations shows that 30S ribosomal proteins S3, S5, S8, S10, S14, S17, S19 and 50S, ribosomal proteins L2, L3, L4, L5, L6, L14, L15, L16, L18, L22, L23, L24, L29, L30 and L36 are all contained in this group. Some other genes, such as translation initiation factor IF-1 is in this group too, which further confirms that this group is related to mRNA translation. It has been observed that most highly conserved operons tend to code protein complexes [20], and COC #6 supports this well because it include highly conserved operons that code proteins for ribosome, which is known to be a large and complex molecular machine, found within all living cells.

### Star-structure COCs and their central nodes

About 22 COCs have one or two central node(s) with most of the other nodes connect to it, which form a star structure. COC #13 (Figure 3C) has such a structure, with central node #4 being adjacent with more than ten nodes. We found that node #4 is an ABC transporter or ABC transporter like protein family, with one exception being a hypothetical protein. While the nodes surrounding it are mainly proteins related to ABC transporter, such as node #1088 and #1072 stand for amino acid ABC transporter permease, nodes #4612 and #4829 stand for polar amino acid ABC transporter inner membrane subunit. In the operons being mapped to COC #13, more ABC transporter related proteins could be found, such as extracellular amino acid-binding proteins and ABC transporter substrate-binding proteins. See more details in File S3. Over all, the main theme of COC #13 is ABC transporter and related proteins, with ABC transporter proteins at the central position, which suggests this kind of protein has a central role in the formation of ABC transporter.

Another star shaped COC #54 is shown in Figure 3D. The central node #11 represents rod shape-determining protein MreB/Mbl; other rod shape-determining proteins MreC and MreD, and some membrane proteins surround it. Interestingly, the DNA repair protein RadC also appears in this COC and has a strong relation with node #11, which suggests some functional relationship between them. See more details in File S3.

Finally, in COC #29 (Figure 3E), there are four paths of length two connecting to node #176 and #206, which are ATP synthase F1 subunit alpha and beta, correspondingly. These surrounding nodes are all ATP synthase subunits gamma, but belong to different OGGs; we suspect they could have similar functions with different mechanisms. All other nodes in this COC are ATP synthase subunits except hypothetical proteins, which could give clues to annotate these genes as ATP synthase related. More details can be found in File S3.

### The genes from biomass degraders and pathogens can be clearly separated in some COCs

Some OGGs are enriched with genes from biomass degraders and some others from pathogens (File S1). In eight specific COCs, these two kinds of nodes can clearly form different paths and are connected by large-degree node(s), hence easily being classified. For example, in COC #27 (Figure 3F), two paths, namely path 1 and path 2, are formed by nodes mainly contain genes from biomass degraders, while path 3 with two nodes contain genes from pathogens. Node #121, connecting these 3 paths, corresponds to nitrogenase iron proteins. In path 3, node #2300 contains protein NifE2, nitrogenase cofactor scaffold and assemble proteins, however, 83% are hypothetical proteins; node #2269 contains NifE1 and nitrogenase vanadium-cofactor synthesis protein VnfN, also 83% are hypothetical proteins. In controversy, genes in paths 1 and 2 are mostly known proteins related to nitrogenase. Such as nitrogen regulatory protein P-II, nitrogenase cofactor biosynthesis protein NifB, molybdenum-iron protein subunit alpha and beta are found in path 1; while nitrogenase molybdenum-iron protein alpha and beta chains are found in path 2; these proteins are not found in pathogens, more details in File S3.

**Figure 4:**
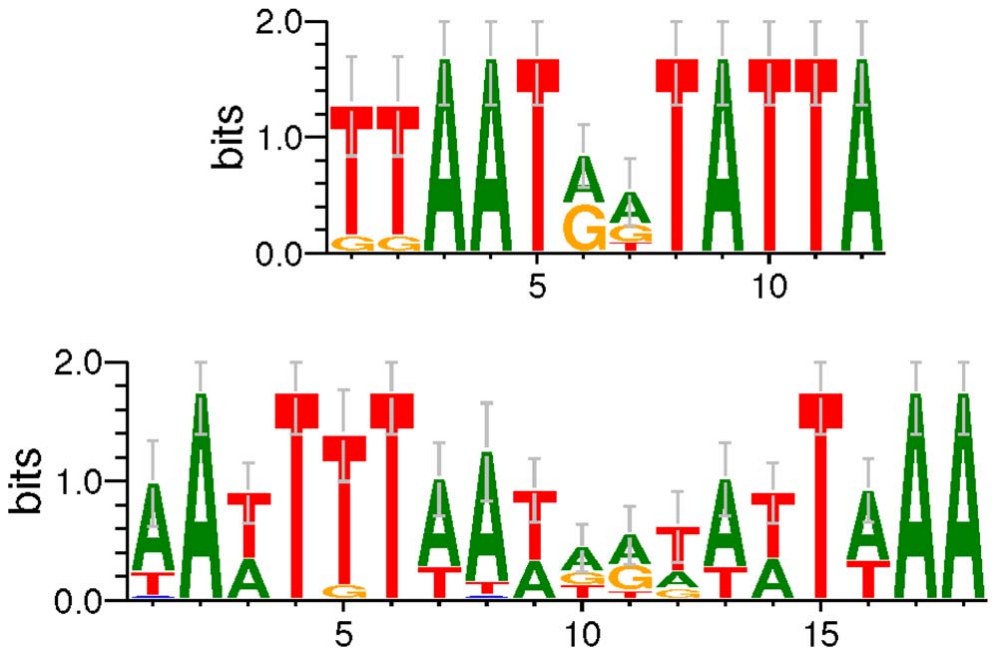
Motifs related to genes in COC 27. The first motif is identified from the promoters of genes in biomass degraders; and the other one is for pathogen.

We suspect that the ability to fix atmospheric nitrogen gas (carried out by nitrogenase) is not as strongly needed in pathogens as in biomass degraders, so some related genes might be mutated or lost in pathogens due to genome reduction [46] during evolution. To infer the regulatory mechanism of these genes, we did *de novo* motif finding for groups of operons (genes) from biomass degraders and pathogens with BoBro2.0 as described in the METHODS section. The most significant motifs from these two groups are shown in Figure 4. The consensus of the motif from biomass degrader is ‘TTAATAATATTA’, and the one from pathogen is ‘AATTTTAATAATATTAAA’; the first is actually a sub-pattern of the second, but with higher information content (9.39 *versus* 5.16). It suggests that the same regulatory mechanism might be adopted by these two groups of genes, but the regulatory sequences are degenerating along with the losing of nitrogenase related genes in pathogens.

## Discussion

Operon structures provide important clues for functional annotation of proteins [9]. However, which genes are placed together in operons varies substantially across bacterial organisms, and recently evolved operons are not suitable for inferring function of genes [7], [47]. In our model, genes are linked by conserved operons from closely related genomes, which provide strong evidence for their functional relationship. Moreover, different linkage patterns could reflect the different roles of the underlying proteins. Overall, our model gives new insights on the organizing principles of genes in operons across closely related genomes and provides valuable clues for elucidating transcriptional regulation and predicting the function of genes.

## Supporting Information

Figure S1 **The largest connected component in the operon alignment graph of the 19 clostridial genomes.**
(TIF)

Figure S2 **Distribution of edge weight in the operon alignment graph.**
(TIFF)

Figure S3 **Distribution of node degree in the operon alignment graph.**
(TIFF)

File S1 **Orthologous gene groups of 19 clostridial organisms.** All the orthologous gene groups are predicted with our in-house orthology identification tool GOST followed by the clustering program MCL.
(XLSX)

File S2 **DAVID functional enrichment analysis for large-degree nodes.** For each node, we pick the gene from *C. thermocellum* as template for the functional analysis in DAVID.
(XLSX)

File S3 **GenBank annotations for selected COCs.**
(XLSX)

File S4 **COC details.** Each COC file contains the nodes, edges and operons, from the 19 genomes, that could be align to this COC.
(RAR)
