# Supplementary figures and images for: Elucidation of Operon Structures across Closely Related Bacterial Genomes
Source: PLoS One. 2014 Jun 24;9(6):e100999. doi: 10.1371/journal.pone.0100999 (PMC4069176; doi:10.1371/journal.pone.0100999)

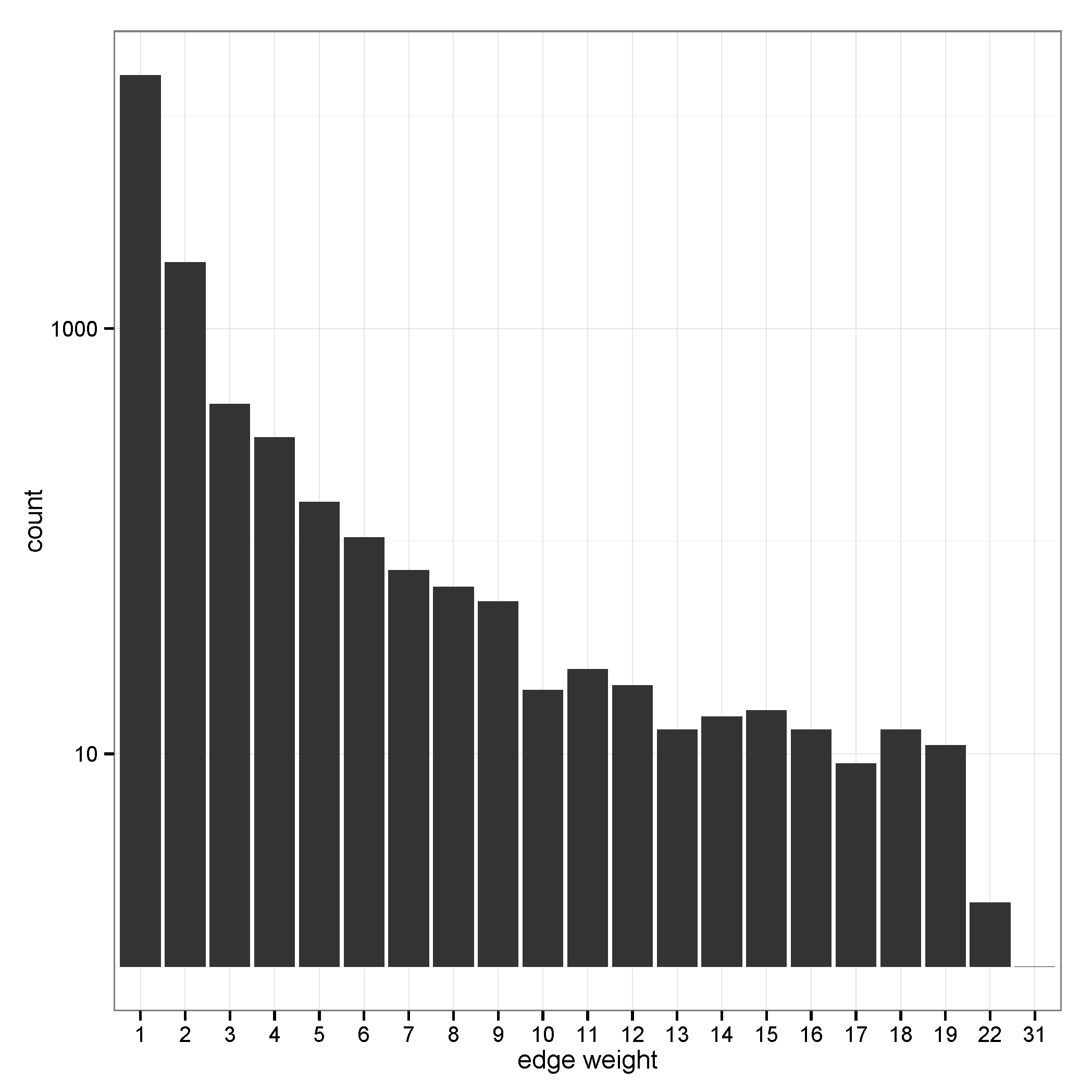

Supplement: Figure S2 — Distribution of edge weight in the operon alignment graph. (TIFF) [file pone.0100999.s002.tif]

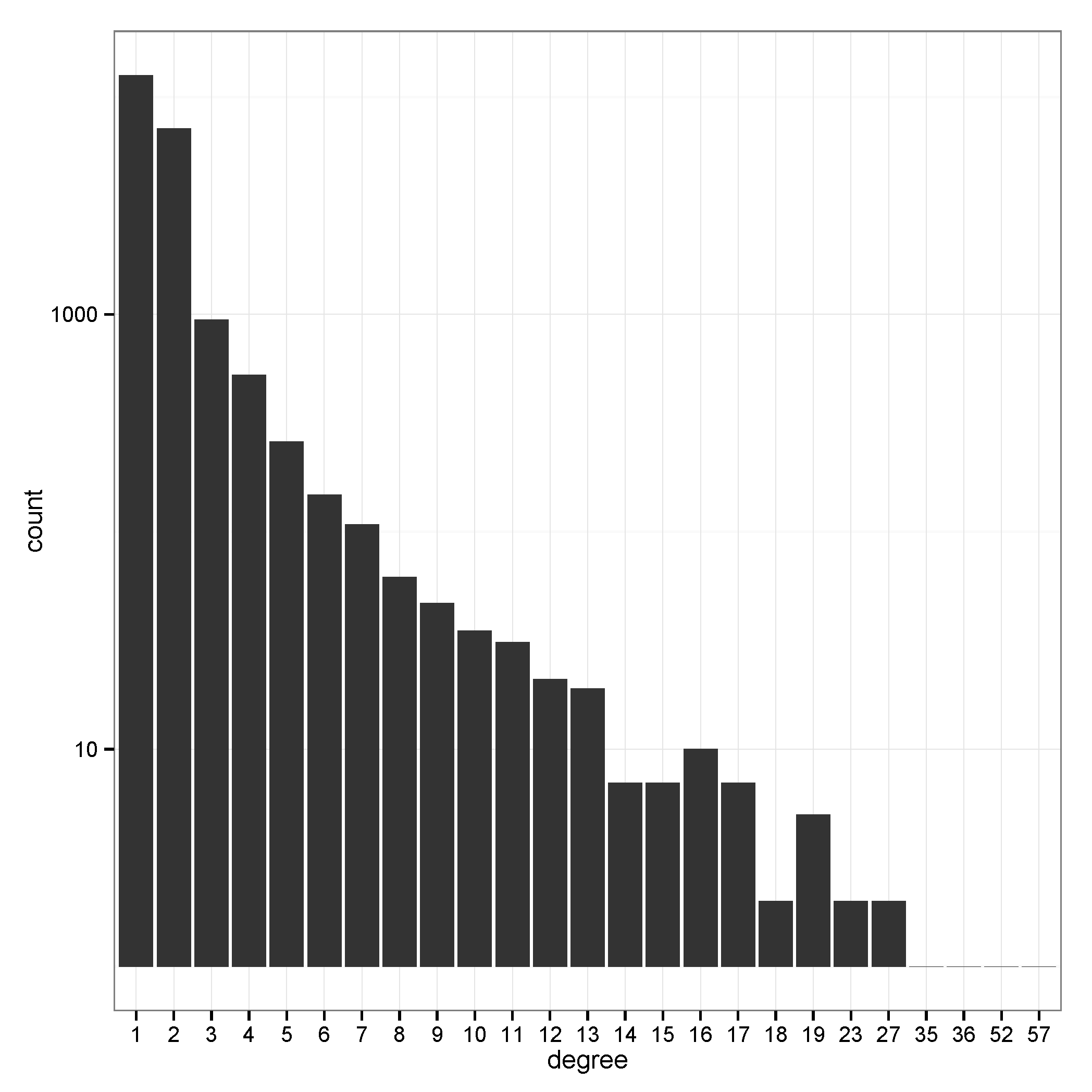

Supplement: Figure S3 — Distribution of node degree in the operon alignment graph. (TIFF) [file pone.0100999.s003.tif]
